# Clinical Anesthesia, South Asian Edition: A Book Review

**DOI:** 10.31729/jnma.7155

**Published:** 2021-11-30

**Authors:** Pawan Kumar Hamal

**Affiliations:** 1 Department of Anesthesiology, Critical Care and Pain, National Academy of Medical Sciences, National Trauma Center, Kathmandu, Nepal

**Keywords:** *anesthesia*, *book review*, *clinical*

## Abstract

Local practices, contextual issues and the therapeutic challenges of South Asian perspectives are generally overlooked in standard Clinical Anesthesia textbooks with more relevance to western scenarios. The South Asian Editors of this book, have made a tremendous painstaking effort to consider these issues and present the content as per the need of the local scenarios in an evidence-based manner.

## BOOK DETAILS

Clinical Anesthesia, South Asian Edition

Adaptation of Eight Edition, 2021

Editors: Paul G. Barash, Bruce F. Cullen, Robert K. Stoelting, Michael K. Cahalan, M. Christine Stock, Rafael Ortega, Sam R. Sharar, Natalie F. Holt

South Asian Editors: Nishkarsh Gupta, Anju Gupta

Publisher: Wolters Kluwer (India) Pvt. Ltd. New Delhi

ISBN Number: 978-93-89859-91-1

## ABOUT THE BOOK

Since the public demonstration of ether in 1846 in Massachusetts General Hospital by William T.G. Morton, anesthesia specialty had made tremendous progress and revolutionized surgical and perioperative care across the globe.^1^ Clinical Anesthesia, a book which was launched in 1990 as a first edition is a popular and textbook of choice all over the world which has taught millions of anesthesiologists in an evidence-based manner and helped them acquire knowledge efficiently. Recently launched eight editions in 2017 follow the legacy and brings a sense of pride among practicing anesthesiologist.^2^ However, it was always realized by the practicing physicians, academicians, and the students of the South Asian countries that the content are more suited to western conditions and seemed less focused on the type of local practices, contextual issues and challenges in therapeutics of South Asian perspectives. Moreover, postgraduate students in this part of the world are on the continuous lookout for comprehensive books adapted to local conditions.

## SOUTH ASIAN EDITION

The South Asian Editors of this book, Dr. Nishkarsh Gupta is a well-known popular academician and gifted teacher along with the colleague Dr. Anju Gupta an upcoming academician from All India Institute of Medical Sciences has made a tremendous painstaking effort to contextualize the book and present the content as per the need of the local scenarios in an evidence-based manner. Care has been taken to maintain the legacy and pride of the late Paul G. Barash, chief editor of this book since its inception in 1990, and also meticulously address the need of the anesthesiologist in a comprehensive way providing services in low- and middle-income countries. The addition of new chapters "Anesthesia for cancer surgeries" and "Palliative Medicine" highlights the changing epidemiology of cancer care in Southeast Asia and prepares anesthesia physicians for providing efficient perioperative care. There were also major updates on chapters of the history of anesthesia, autonomic nervous system, anatomy and physiology, common monitoring techniques, airway management, neuraxial anesthesia, anesthesia for cardiac surgery, vascular and endovascular surgeries, obstetric anesthesia, chronic pain, critical care, neonatal anesthesia, pediatric anesthesia, anesthesia for orthopedic surgeries, and cardiopulmonary resuscitations. The chapters on anesthesia for cardiac surgery, chronic pain, neonatal anesthesia, pediatric anesthesia, anesthesia for vascular and endovascular surgeries are great to read and thorough.

Efforts have been made to incorporate the use of ultrasound techniques in different chapters particularly regional and peripheral blocks, airway evaluation, critical care to highlight the innovative technique for enhancing better perioperative care. There is a great improvement in illustrations, tables, images, algorithms in most of the chapters to provide current knowledge in anesthesia care. The contents have been incorporated with online videos that are extremely useful for better understanding in the present context.

Addition of "Must Know" and "Desirable to know" content in the start of each chapter and pertinent multiple-choice questions at the end can be very helpful for postgraduate students who are preparing for final and competitive exams.

## WAYS FORWARD

The book is the first effort of both the south Asian editors and it is yet to be seen from the readers whether the book has met its objectives. However, it can be said with confidence that this book will meet the expectation and will evoke the sense of pride and belonging among the anesthesiologist in this part of the world.
